# Fiscal Integration in an Experimental Union: How Path‐Breaking Was the EU's Response to the COVID‐19 Pandemic?

**DOI:** 10.1111/jcms.13246

**Published:** 2021-09-05

**Authors:** Waltraud Schelkle

**Affiliations:** ^1^ London School of Economics and Political Science London

## I Inauspicious Start and Surprising Turnaround

The Covid‐19 pandemic could not have started worse for the EU (Herszenhorn and Wheaton, 2020). In late February 2020, Italy requested supplies of medical equipment from other member states under the EU's crisis response programme, the Civil Protection Mechanism. The Commission duly escalated it to highest alert. But there was no response. Alarmed by Italy's request, France engaged in a stock‐taking exercise that did not allow trading. Czechia, Germany, and Poland introduced export controls for medical equipment. Embarrassingly, China, Cuba and Russia came to the rescue more readily than fellow‐Europeans (Poggioli, 2020). Under pressure from the Commission, German controls were lifted soon after (Reuters, 2020), but support for EU membership in Italy plummeted. The prospects for collective, forward‐looking crisis management were bleak. Considerable policy resources had been spent since 2008 and their replenishment did not look promising. Some interventions, especially of the Troika, had done lasting political damage. A deepening political crisis seemed imminent, dividing an already divided union even further.

Yet, over several months, the EU managed to agree on a massive support package for the anticipated long slow recovery. The breakthrough came in mid‐July. This was not a Hamiltonian Moment, founding the fiscal union that some see as the panacea for all Euro Area (EA) woes (Kaletsky, 2020). But the flagship Recovery and Resilience Facility (RRF) recognizes that a preventive fiscal capacity is required to shield and support the EA's most vulnerable members. This is in stark contrast to the European Stability Mechanism (ESM), which handed out cheap rescue loans only once a government was shut out of bond markets, and imposed intrusive prescriptions for fiscal policy and institutional reforms, overseen by the Troika. The RRF adopts the principle that the promise of mutual support can prevent a panic before it spreads. This subsequently changed the political economy of Covid‐19, although the public relations debacle of the Commission's vaccine procurement and the slow roll‐out of the immunization programme in member states continued to test political unity.

This article analyses what made the dynamic of the Covid‐19 pandemic so different in political‐economic terms. I look at this dynamic through the lens of three significant reforms, which were accompanied by a new crisis politics in which heads of state directly addressed sceptical audiences in other countries as well as their own. I provide evidence that the legacy of failure in the EA crisis spurred attempts by major protagonists to break with a path that they saw as politically disastrous for the EU polity as a whole.

This should surprise any long‐term observer of EU affairs. Ever since its revival in the late 1980s, the EU has enshrined long‐term commitments of its members to openness and stability (Majone, 1996). The union operates in a rule‐bound, path‐*dependent* way that militates against such turnarounds. This scholarship is still valid and can actually explain why fiscal integration remains difficult even though a majority of member states has already given up their national currency. I propose a historical‐institutionalist argument in the tradition of Pierson (2004) that can explain potentially path‐breaking (or switching) reforms in the evolution of the experimental EU polity. The argument draws on literature on the possibility, and nature, of change in path‐dependent institutional development (Streeck and Thelen, 2005; Capoccia and Kelemen, 2007). The reforms of 2020 can be seen as a critical juncture in a longer process, in which the EU transcends the confines of a regulatory polity but does not commit to fiscal federalism either (Genschel and Jachtenfuchs, 2014; Kriesi *et al*., 2021, pp. 4–6).

The next section reviews how intergovernmental contestation over the legacy of the EA crisis led to a surprising political response to the Covid‐19 pandemic. The subsequent section explains the innovations in the recovery packages agreed at the EU level. The last section outlines how we can understand path‐breaking in the EU polity theoretically.

## II A Surprising Response to a Multi‐Dimensional Crisis

After the inauspicious start, political actors tried to shape the public conversation about the pandemic. This conversation revolved, first, around crisis interpretation: is the crisis a shock from outside the EU or is past mismanagement to blame; does it affect all or primarily vulnerable Southern Europe members? The other debate concerned the appropriate economic response by the EU: how much additional support from the EU is needed to rekindle growth in member states?

For answers, I look primarily at the public statements of five heads of state, using discourse as evidence for a political strategy of change (Schmidt, 2000). It was one of the notable features of pandemic politics that political leaders spoke directly to audiences across borders where they expected opposition. The selection of these five leaders reflects the focus of media reports on the EU's pandemic management. There are, first of all, the Prime Ministers of Italy, Guiseppe Conte, and of Spain, Pedro Sánchez, who started the conversation by calling for bold action to a pandemic that hit them first and hard. Mark Rutte, the Dutch Prime Minister, was their main opponent, representing at least three more member states (Austria, Denmark, Sweden). French President Emmanuel Macron (France) joined Italy and Spain in an initiative of nine countries calling for Eurobonds in late March and adopted the role of an interlocutor. The German Chancellor Angela Merkel kept a low profile until mid‐May when she and Macron proposed a surprisingly big transfer scheme, providing an anchor for the grand bargain under the German Council Presidency in July.

### III What Kind of Crisis?

An early influential diagnosis can be gleaned from a transnational statement by Prime Minister Sánchez, after the first extraordinary European Council on 10 March, 2020. More clearly than the Council conclusions, Sánchez stressed the dual nature of the crisis: a global emergency with a health and an economic dimension (Sánchez, 2020). This proved to be consequential as it divided the pandemic into a fast‐burning public health crisis and a slow‐burning recovery crisis (Seabrooke and Tsingou, 2019). While member states and the Commission started to coordinate their public health responses from mid‐March onwards, governments could afford some time to consider measures addressing the economic fallout (Truchlewski *et al*., 2021). Conte (2020a) seconded Sánchez a week later in an interview with the FT, stressing that this was a global, not a Southern European, emergency.

An open letter to Council President Michel signed by nine member states 1 was based on this distinction but had a normative thrust: ‘we are all facing a symmetric external shock, for which no country bears responsibility, but whose negative consequences are endured by all’ (Wilmès *et al*., 2020). This letter, published on 25 March, would become notorious for its demand for a joint debt instrument (‘Coronabonds’). The signatories included Greece and Ireland, Luxembourg and Slovenia, which had in fact very different experiences of the pandemic. Hence, this letter was a call for solidarity, directed against the narrative of the EA crisis in which those countries that needed a bailout were blamed for past failings that justified onerous corrections.

The interpretation ‘symmetric shock, external cause, affecting all’ was specifically directed against the Dutch government. In late March, Finance Minister Hoekstra scandalized Southern Europeans when he reportedly asked for an investigation into why Spain was fiscally not prepared for the pandemic (Euractiv, 2020). The Portuguese Prime Minister called his remarks ‘repugnant’. A dozen mayors of Italian cities posted a one‐page letter as an advertisement in a conservative German newspaper on 31 March, which was widely circulated in Dutch social media. In this letter to ‘dear German friends’, the Dutch position is denounced as a prime ‘example of a lack of ethical standards and solidarity’. The Dutch government was accused of raiding other countries' tax bases to the detriment of the poorest (Calenda, 2020, my translation). Simultaneously, Conte gave extensive interviews in the Dutch newspaper *De Telegraaf* and the German weekly *Die Zeit*, stressing that the pandemic resembles a natural catastrophe and ‘Dutch citizens may need guarantees, too’ (Conte, 2020b; Google translation). Hoekstra was shamed into an apology.

The symmetric character of the pandemic could be framed, however, as a reason for why nobody should make extraordinary demands on others. Three weeks before the July summit, Mark Rutte stated in an interview with an Italian weekly: ‘We owe solidarity to the countries most affected by the pandemic, knowing, however, that we too have been seriously affected. This means that states that need and deserve help must also ensure that in the future they are capable of dealing with such crises on their own in a resilient way.’ (Valentino, 2020, Google translation) This understanding of resilience made it into the name of the recovery fund and it stands for reform in return for grants.

In her first public statement to prepare Germany's rotating Council Presidency, Merkel (2020) adopted a multi‐faceted diagnosis. It distilled the agreeable essence out of the dissenting positions. The interview, simultaneously published on 20 June in six European newspapers, 2 started with what the Dutch‐led coalition stressed: ‘time and again it has been shown that Europe is not yet sufficiently resistant to crises’. But she also highlighted the point Southern European leaders had made: ‘the coronavirus pandemic is confronting us with a challenge of unprecedented dimensions. It has struck us all indiscriminately’. And she expressed the shared fear of a populist backlash: ‘Very high unemployment in a country can become politically explosive and thereby increase the threat to democracy. For Europe to survive, its economy needs to survive.’ Merkel's speeches in 2020 expressed repeatedly the quintessential liberal concern that representative democracy depends on economic stability.

Merkel did not hide Germany's interest in the integrity of the Single Market. The Coronabonds letter had stated this appeal to enlightened self‐interest early on: ‘Preserving the functioning of the Single Market is essential to give all European citizens the best possible care and the strongest guarantee that there will be no shortage of any kind.’ (Wilmès *et al*., 2020) Border closures, the disruption of supply chains, and the uneven ability to benefit from the easing of state aid rules were in nobody's interest. The threat to mutually beneficial economic interdependence became the compromise diagnosis, to which all sides could sign up.

### IV What Kind of EU Support?

The debate on what response should follow from the diagnosis was dominated by the question of what role the ESM should play. Governments changed their positions as lternativesto the ESM appeared. Coronabonds were countered with limited grant proposals. It took the ‘innovative’ financial design of the recovery fund to set a path to agreement.

In an early FT interview (19 March), Conte (2020a) echoed ECB President Lagarde (2020) when he stated:

Monetary policy alone cannot solve all problems; we need to do the same on the fiscal front and [..] time is of the essence [..]. The route to follow is to open ESM credit lines to all member states to help them fight the consequences of the Covid‐19 epidemic, under the condition of full accountability by each member state on the way resources are spent.

But the domestic coalition of opposites that Conte headed since 2018 was less conciliatory: both the right‐wing Lega and the Five Star Movement were fiercely opposed to taking recourse to ESM loans since the ESM Treaty (Article 3) requires ‘strict conditionality’ for any support. Lega leader Salvini portrayed conditionality as ‘an attack on our country’ by ‘loan sharks in Berlin and Brussels’ (Khan, 2020).

By early April, after the Coronabonds letter had been published, Conte argued that precautionary ESM credit lines were not up to the task. The sensibilities that had made Conte take this turn were acknowledged by Finance Minister Scholz in an interview with German media, although he supported the activation of the ESM: ‘There won't be any senseless conditions as there were sometimes in the past. No troika will come into the country to tell the government how to do politics. This is about support in the crisis.’ (Barfield, 2020) This last phrase acknowledged that in the EA crisis, the ESM had acted primarily as a ‘firewall’ for the guarantor countries; the term is often used in official documents..

The Dutch Prime Minister, by contrast, made it clear that his first preference would be for the well‐off members making ‘a gift’ to hard hit countries (in the order of €1bn from the Netherlands). If the ESM had to be used at all, then only with the usual conditionality of structural reforms attached (Khan, 2020). Charity rather than solidarity was the gist of Rutte's proposal. Interestingly, he had implied that grants were a preferable option to loans before this was publicly discussed.

Crunch time came at a Eurogroup meeting on 8–9 April. A 14‐hour all‐night tele‐conference ended in a standoff (Khan, 2020). The Italian and the Dutch finance ministers had clashed; the former asked for assurance that the conclusions would mention ‘debt mutualisation as a tool for the economic recovery’ and ESM loans would have no conditionality attached. Hoekstra categorically rejected both demands, with reference to a consensus in the Dutch parliament and his coalition government. And yet, on 9^th^ April, the Eurogroup delivered its report on the comprehensive response to the pandemic. It contained, inter alia, crucial details of the ESM Pandemic Crisis Support which was signed off by all relevant bodies about a month later (European Council, 2020a: para. 16). Debt mutualisation was not explicitly mentioned. How was this possible? Khan (2020) reports where negotiations had broken off: ‘“Coronabonds has to die if we are to get a compromise,” said one northern eurozone diplomat. A draft circulating in the early hours last night mentioned the use of “innovative financial instruments” — too much for the Dutch and too little for Italy.’ In fact, it was enough for both. The package deal was that innovative financial instruments were to replace Coronabonds. The battle was now on for these instruments.

Shortly before the Eurogroup meeting, two liberal MEPs, Luis Garicano and Guy Verhofstadt, had proposed just such an innovation, a ‘European Reconstruction Fund’. 3 On 15 April, the European Parliament passed a resolution (2020: para. 17) with the votes of the four mainstream party groupings ‘calling on the European Commission to propose a massive recovery and reconstruction package [..], beyond what the European Stability Mechanism, the European Investment Bank and the European Central Bank are already doing, that is part of the new multiannual financial framework (MFF); [..] the necessary investment would be financed by an increased MFF, [..] and recovery bonds guaranteed by the EU budget; this package should not involve the mutualisation of existing debt and should be oriented to future investment.’ A day later, the Commission President gave a speech in which she endorsed the novel idea: ‘The European budget will be the mothership of our recovery.[..] We will use the power of the whole European budget to leverage the huge amount of investment we need to rebuild the Single Market after Corona.’ (Von der Leyen, 2020).

Based on the liberal MEPs' idea, the Socialist finance minister of Spain, Nadia Calviño, proposed a €1.5tn reconstruction fund on 19 April. It would provide grants financed by perpetual bonds on which the EU would have to pay interest only. It sounded too daring. Yet, a month later von der Leyen presented technical details on the recovery fund of unknown quantity to the European Parliament. This was five days before the Franco‐German proposal endorsed the principle of bond finance guaranteed by the EU budget for grants only. However, it proposed a transfer volume of €500bn, to be repaid in the long term through the Commission's ‘own resources’. As the Commission has no ‘own resources', this must mean finding a source of tax revenue, although this cannot be said explicitly as it would require a Treaty change. Ten days later, the Commission proposal added to this €250bn in loans. It was dubbed ‘Next Generation EU’ and eventually combined a number of smaller programmes with the RRF of €672.5bn (of which €312.5bn are grants).

On the evening before the Commission proposal, the ‘Frugal Four’ coalition presented a non‐paper for an ‘Emergency Recovery Fund’. They reiterated the ESM principles of ‘loans‐for‐loans’ 4 and strict conditionality. But they accepted the principle of allowing the Commission to borrow within the multi‐annual budget (Non‐paper, 2020). This created an issue linkage between the recovery fund and budget negotiations that could be turned to their advantage. The paper was not dated and not signed, but posted on the Dutch government's website. It reads like a last‐minute reminder that the four countries would have to be compensated for agreeing to anything resembling the Franco‐German proposal. Compensation came in the guise of generous rebates that reduce their net contributions to the EU budget.

The Eurogroup meeting in early April had started the deliberations on the grand bargain of the EU budget and a recovery fund. The Franco‐German proposal was the political signal of an emerging compromise. Merkel said as much at a pre‐summit press conference with Italian Prime Minister Conte in mid‐July: ‘it is important [..] that [the recovery fund] is something massive, something extraordinary. [..] It has to be a special effort to show that Europe wants to be united; there is a political dimension beyond the numbers.’ (Conte and Merkel, 2020, my translation) In line with Merkel's desire for a unifying gesture, the fund would apply to all EU members. This also served her goal of preventing Eurobonds that would have provided funding only to euro area members.

In contrast to the other two reforms, the ‘temporary Support to mitigate Unemployment Risks in an Emergency’ (SURE) was a swift and uncontroversial affair. Throughout March, member states announced massive support programmes that dwarfed their fiscal responses during the financial crisis in 2008–09 (Bruegel, 2020). Within these fiscal stimulus programmes, country after country announced relatively generous job retention schemes, obviously in the expectation that lock‐downs would not last for very long. Governments in OECD countries supported around 50 million jobs, ten times as many as during the financial crisis (OECD, 2020). A re‐insurance scheme for national unemployment benefits has been discussed in EU circles for a while (Vandenbroucke *et al*., 2020). A job insurance scheme based on low‐interest‐rate loans was easier to agree since it is inherently temporary. Unlike unemployment insurance, short‐time and furlough schemes are not permanent programmes in national welfare states. Hence, the Commission was able to propose SURE very quickly, on 2^nd^ April, and put it into effect on 19^th^ May, 2020. It had a volume of €100bn, of which €75.5bn were disbursed by end‐of‐March 2021 (European Commission, 2021). It seemed generous at the time although less so after a third wave of the pandemic rolled over the continent.

## V The Innovation of Three Reforms

How path‐breaking or path‐dependent were these reforms in institutional terms? Unlike many political structures, EU institutions do come with an instruction sheet, to paraphrase Blyth (2003). But political agency and historical contingencies can alter the original intentions significantly. Whether the intended innovations will materialize in years to come depends on institutional practice.

In chronological order of coming into effect, the ESM Pandemic Crisis Support was first and explicitly modelled on the ESM's Enhanced Conditions Credit Line. But under the latter programme, the ‘requesting country has to sign a Memorandum of Understanding (MoU) detailing policy conditionality, aimed at addressing the remaining weaknesses’ (ESM, 2020). The Pandemic Support provides support on demand of a state government, each of which is entitled to loans worth 2% of its GDP. Above all, no conditionality is imposed beyond earmarking, which requires spending to be pandemic‐related, detailed in a Pandemic Response Plan (PRP), which is standardized for all ESM members. 5 The overriding purpose was to remove the stigma attached to ESM loans.

The reform as such is not innovative, indeed it ostentatiously continues an existing credit line of the ESM. But it stands for an explicit coordination of fiscal and monetary policy in that it builds a bridge between Quantitative Easing by the ECB and Troika lending to governments. The ECB can buy government bonds from banks only after a prescribed time lag, so as not to violate the prohibition of public debt monetisation under Article 123 TFEU. This means that, if financial investors shun a government's new bond issue, the ECB cannot directly step in the bank has already hovered up all qualifying bonds. Previously, such a government could only get a Troika programme. Now, it can avoid acute liquidity problems arising from the loss of market access by drawing on Pandemic Support, without the strict conditionality and lengthy negotiation of an MOU that accompanies other precautionary ESM credit lines with similar purposes.

SURE, the EU's re‐insurance programme for national job‐retention schemes is a relatively large labour market stabilizer, to the tune of close to 1% of EU‐GDP in 2020. It encouraged member states to introduce such schemes and thus maintain employment, substituting for unemployment benefits as automatic stabilizers. Job retention schemes subsidize labour hoarding in existing businesses instead of down‐sizing businesses through unemployment, allowing firms to use this time for training staff and adopting new work practices. Moreover, SURE could be used for schemes to support the self‐employed, who are typically not entitled to unemployment benefits. Loans under SURE operate like an overdraft for national expenditure on job (and business) maintenance. There is no conditionality attached, beyond the member state providing evidence of costs of job retention incurred. The Commission must report back to the Council every six months on how loans have been used (Regulation 2020/672, Article 14). The programme is financed out of the EU budget but secured by explicit guarantees from member states, given that it exceeds the margin (‘headroom’) for excess demand on budget resources (Garicano, 2020).

SURE is innovative in that it has no obvious predecessor in other EU programmes. Re‐insuring job retention marks a stark contrast to EU interventions in the protracted crisis since 2008 where the maxim of structural reforms was to flexibilise labour markets. The EU's official labour market paradigm is flexicurity (European Commission, 2007), that can be summarized as ‘protecting workers, not jobs.’ Job retention goes directly against higher labour market turnover. At the same time, the adjective ‘temporary Support’ emphasizes that SURE is a programme for an extraordinary situation. If SURE became a permanent re‐insurance scheme for employment and unemployment schemes in the EU, it would mark a paradigm shift towards reinforcing national automatic stabilizers rather than prioritising fiscal retrenchment that switches off automatic stabilizers.

Finally, the grant element of the RRF has affinity with a gigantic cohesion programme. Grants are allocated such that they help poorer member states more but also those harder hit economically by the pandemic, i.e. the allocation combines redistribution with insurance. 6 The grant element also required giving the Commission for the first time the power to tax. The Council authorized the Commission ‘to borrow on behalf of the Union in capital markets’ (European Council, 2020b, A.3). The long‐term bonds are secured by the budget itself, skilfully avoiding the dead‐end of the Eurobonds discussion. The RRF is procedurally tied to the European Semester (Regulation 2021/241: Preamble para 17), for the first time providing financial incentives for compliance with this process of coordinating economic policies. The European Semester also ensures that the European Parliament has a say; the legislators' insistence on the rule of law as a precondition for funding signalled that MEPs are keen to use this power.

Conditionality is minimal (and the term is avoided): payments can be suspended if recommendations to correct an excessive deficit have not been followed twice (Regulation 2021/241: Article 10). This is a minimal condition because the RRF itself should make it easier to meet the fiscal rules. Recovery and resilience plans must set out a package of reforms and public investment projects, to be implemented by 2026; over half of the requested finance must be spent on climate change and digital transition. The Commission assists in drawing up these plans to a presentable standard and the Council signs them off on a case‐by‐case basis. Obviously, the latter allows the sceptics in the Council to put pressure on recipient governments.

The RRF is a novel support mechanism that gives fiscal powers to the EU level that it did not have before. The Commission was allowed to ‘mortgage’ the EU budget in order to resist both Coronabonds and ever more contingent liabilities (guarantees) for overstretched national budgets (Garicano, 2020). Providing grants on a macroeconomic scale is another novelty and an element of fiscal federalism. Finally, member states have now an incentive to give the Commission the power to tax, a power which is likely to be exercised through a digital tax and environmental levies, which are sources that nation‐states find hard to target. These are big steps and yet incremental insofar they are reversible. The RRF is a temporary measure and it applies to the EU as a whole; the link to the common currency is absent as long as not all EU members adopt the euro. The powers to tax and spend are earmarked for purposes of fighting the pandemic. Its conspicuously redistributive design makes containment and reversal somewhat more likely because better‐off countries have strong incentives to pay close attention to the (mis‐)use of funds by beneficiary members.

The three reforms constitute fiscal integration for an experimental polity. The EU polity provides targeted re‐insurance when catastrophe or systemic destabilization overwhelms national risk pools (Schelkle, 2017, pp. 316–22). The RRF re‐insures asymmetric budget risks and SURE re‐insures relatively short, sharp shocks to businesses. The ESM Pandemic Crisis Support re‐insures liquidity risks for sovereigns and thus prevents solvency problems. The political wisdom of these reforms lies in the fact that they allow EU governments to sit on the fence for longer as regards a common budget while providing the politically and economically called‐for stabilizers. Charting a way to closer fiscal integration without firmly committing to a divisive fiscal federation was arguably the only viable decision for a union of democracies.

## VI A Historical‐Institutionalist Explanation of Path‐Breaking Reform

The change we have seen in the EU's latest crisis is of wider theoretical relevance. The process and the outcome of the ESM reform and massive recovery funding can be understood as a deliberate attempt to change the path of bailout funding. This path had been paved with a series of Troika programmes since 2010 and, de jure, tightened fiscal rules. They constituted a legacy of perceived failure as public statements by Conte, Macron and Sánchez made clear. Historical institutionalism can explain why established paths and legacies require deliberate, often futile, attempts at moving beyond them. Incremental change is the expected pattern of institutional evolution (Streeck and Thelen, 2005). But knowing this, actors may also try to construe crises as critical junctures in which the future is open and agency can have a disproportionate impact on the course of events (Capoccia and Kelemen, 2007, pp. 351–2; Grzymala‐Busse, 2011, p. 1277).

The experimental polity of the EU lends itself to such situations. 7 Its dispersed authority empowers networks of national and supranational executives to make decisions that can change the direction of what in normal times proceeds slowly through highly codified, regularised and routinised policy processes. In this densely institutionalized polity, agents are accustomed to seizing opportunities thrown up by a massive policy challenge, and presenting them as critical junctures in which accelerated change can happen (Capoccia and Kelemen, 2007, p. 358). Fiscal integration, a major construction site of this polity, has repeatedly shown this pattern.

The publication of the Coronabonds letter is arguably evidence for such political agency. After all, proposing an institutional reform that would require lengthy negotiations was hardly an obvious response to an emergency. The pandemic was also not confined to euro area members. But the letter demonstrated the determination, especially of Italy's and Spain's governments, of resisting a replay of euro area crisis management and reverting to ESM programmes as the default option. The publication of a proposal for €1.5trn worth of grants by the Spanish government indicated that small would not do to satisfy the promise of innovative financial instruments when Coronabonds were taken off the table. The refusal by Italy's Prime Minister to accept standard ESM treatment told those on the safe side of the firewall that they required Italy's cooperation if they wanted protection.

Following the trodden path was a distinct possibility, which is compatible with the notion of a critical juncture (Capoccia, 2015: 151). The Frugal Four, above all the Dutch government, volunteered to play the conservative force that defended a no‐change scenario. But they were constantly on the back foot. Their progressive opponents not only drove the policy discussion but, above all, engaged in open‐letter and interview diplomacy that captured citizens' sympathy for the plight of their European neighbours. A week after the Coronabond letter and a day after the Italian mayors' letter, the German Bild‐Zeitung had on its front‐page an open letter to Italian citizens. It assured them that 'We are with you! We weep with you [..]. Without you, the German economic miracle would not have been possible.’ (Bild, 2020, my translation) Readers also got a translation into Italian. This from a tabloid not known for a sympathetic view of Greece's suffering during the euro crisis.

A decisive contingent factor that worked against the status quo was Germany's turn in the rotating Council Presidency, under a Chancellor who must have been concerned about her legacy. *Ferrera et al*. (2021) show in detail how Merkel used her favourable polling during the early phase of the pandemic to engage in an exercise of ‘polity‐maintenance’. Repeatedly, she made the case to domestic audiences that this was a crisis that warranted Germany's solidarity with other EU members, not only for its own economic good but also because a united Europe remains the foundation of the German state.

Even if, in the long‐run, these reforms do not amount to a decisive permanent step to further fiscal integration, they still mark a critical juncture in 2020. They would have shown an alternative, namely targeted re‐insurance of macro‐risks in the EU as a whole, as a potential diversion from the path‐dependent development of the EA (Capoccia, 2015, p. 160). I have indicated that the ESM and even the RRF reform have precedents and can revert to the status quo ante. But political awareness of path‐dependence can also stimulate strategies that are the undoing of historical determinism. Here, this meant that the crisis narrative had to be changed and that the contestation over the appropriate response had to be forward‐looking and pre‐emptive. This shows understanding of a basic historical‐institutionalist insight, that default options are privileged simply because they are readily available and have already established stakeholders. Blame games then tend to add insult to injury and do lasting political damage. It was the good fortune of the EU this time that just when two Southern European countries were brought again to their knees by a crisis, their governments refused to let the shadow of the past dictate the future.

## Footnotes

^1^
Heads of state from Belgium, France, Greece, Ireland, Italy, Luxembourg, Portugal, Slovenia, and Spain.

^2^
The Guardian, Le Monde, La Stampa, La Vanguardia, Polityka and Süddeutsche Zeitung; quotes are from the Guardian transcript.

^3^
A first version circulated on 2 April, published in a popularised form as a Project Syndicate blog on 15 April. See Garicano (2020) for links to relevant documents.

^4^
That is, the ESM raises funds in bond markets to finance credit for programme countries.

^5^
Non‐EA members can become ESM members if they sign the Fiscal Compact.

^6^
Allocation criteria are based on population size and the inverse of GDP level plus (for 70% of amount) relative unemployment rate in 2020–21 and (for 30%) change in real GDP in 2020–21 (grants (Article 11 of Regulation 2021/241, Annexes 1–3).

^7^
Kriesi *et al*. (2021) provide a fuller exposition of the underlying theory. The term experimental union goes back to Laffan *et al*. (1999).
